# Micromegas with GEM preamplification for enhanced energy threshold in low-background gaseous time projection chambers

**DOI:** 10.12688/openreseurope.19258.1

**Published:** 2025-02-18

**Authors:** Juan Francisco Castel Pablo, Susana Cebrián Guajardo, Theopisti Dafni, David Díez Ibáñez, Javier Galán Lacarra, Juan Antonio García Pascual, Álvaro Ezquerro Sastre, Igor García Irastorza, Gloria Luzón Marco, Cristina Margalejo Blasco, Héctor Mirallas Sánchez, Luis Obis Aparicio, Alfonso Ortiz de Solórzano, Óscar Pérez Lázaro, Jorge Porrón Lafuente, María Jiménez Puyuelo

**Affiliations:** 1 Center for Astroparticles and High Energy Physics (CAPA), University of Zaragoza, Zaragoza, Aragon, Spain

**Keywords:** Dark Matter, WIMPs, Time Projection Chamber, Micromegas, Underground Science, Low Background Techniques, Radiopurity

## Abstract

**Background:**

We develop the concept of a Micromegas readout plane with an additional GEM preamplification stage placed a few millimetres above it to increase the maximum effective gain of the combined readout.

**Methods:**

We implement it and test it in realistic conditions for its application to low-background dark matter searches like the TREX-DM experiment. For this, we use a Micromegas of microbulk type, built with radiopure materials.

**Results:**

We report on GEM effective extra gain factors of about 90, 50 and 20 in 1, 4 and 10 bar of Ar-1%iC _4_H _10_. These results are obtained in a small test chamber allowing for systematic scanning of voltages and pressures. In addition, a TREX-DM full-scale set-up has also been built and tested, featuring a replica of the fully-patterned TREX-DM microbulk readout.

**Conclusions:**

The results here obtained show promise to lower the threshold of the experiment down to 50 eV _*ee*_, corresponding to substantially enhanced sensitivity to low-mass WIMPs.

## 1 Introduction

Gaseous time projection chambers (TPCs) are versatile detectors that can measure the energy and track of ionizing particles in three dimensions. They have been widely used in various fields of physics, such as high-energy physics, nuclear physics, astroparticle physics and medical imaging ^1^. One of the main advantages of TPCs is their ability to discriminate different types of particles based on their energy loss and track shape, which is crucial for reducing backgrounds in rare event searches. Modern incarnations of TPCs include micro-pattern gaseous detectors (MPGDs) as sensing planes. Example of these are the MICRO-MEsh GAseous Structure (Micromegas) ^2^ and Gas Electron Amplifiers (GEM) ^3, 4^.

Micromegas is a type of MPGD that consists of a thin metallic mesh placed O(50) µm above a segmented anode. The gas volume between the cathode and the mesh acts as a drift region, while the gas volume between the mesh and the anode acts as an amplification region. The electric field in the amplification region is much higher than in the drift region, creating an avalanche of electrons that induces a signal on the anode ^2^.

In Micromegas of the “microbulk” type, the whole amplification structure is produced by chemical processing of a double-sided copper-clad polyamide laminate, onto which the mesh and the anode pattern are etched ^5^. This type of detector is particularly well suited for low-background experiments thanks to its low intrinsic radioactivity ^6^. Indeed, it has been intensely developed in dedicated R&D projects ^7, 8^ and is now being used in solar axions ^9, 10^, neutrinoless double beta decay ^11^ and direct dark matter searches ^12– 14^.

Although achieving higher signal-to-noise ratio is always a desirable property for every application, low detection threshold is especially relevant in dark matter experiments aiming at the detection of the low-energy nuclear recoils produced by the collisions of galactic WIMPs with target nuclei. In these experiments, the spectral distribution of the signal concentrates at low recoil energy, exponentially decaying for higher values ^15^. For experiments specifically targeting low-mass WIMPs, lowering the detector threshold is an important line of detector development, that automatically translates to better sensitivity, while allowing access to lower WIMP masses.

The intrinsic signal amplification happening in the Micromegas gap of the TPC, effectively decoupling the detector threshold from the total size of the TPC, is one of the appealing features that motivates the application of this technology in the TREX-DM experiment. TREX-DM ^12^ is a high-pressure, low-background, Micromegas-based TPC looking for low-mass WIMPs in the Canfranc Underground Laboratory under the Spanish Pyrenees. The TREX-DM TPC has been designed to have an active volume of 20 L, which translates into 0.32 kg of Argon mass at 10 bar (or, alternatively, 0.16 kg of Neon). It is composed of a cylindrical vessel made of radiopure copper, with a diameter of 0.5 m, a length of 0.5 m and a wall thickness of 6 cm. These dimensions are set by the requirements that the vessel holds up to 10 bar(a) of pressure, while at the same time constitutes the innermost part of the shielding. The vessel is divided into two active volumes by a central mylar cathode, which is connected to high voltage by a tailor-made feedthrough. At each side there is a 16-cm-long field cage defined by a series of copper strips imprinted on a kapton substrate supported by four PTFE walls. At the two ends of the active volumes, two 25×25 cm ^2^ squared microbulk Micromegas readouts are placed as sensing anodes, each of them patterned with ~ 1 mm pixels, interlinked with 512 strips in an *x – y* layout.

Especially optimized Micromegas test set-ups have shown that very high gains, of even >10 ^6^, are achievable ^16^. However, the constraints imposed by the environment of a real experiment (the need for stable operation over long periods, robustness of operation, total absence of destructive discharges, large area, large readout segmentation, a controlled level of electronic noise, a given gas composition and pressure determined by physics, etc.) means that an energy threshold only somewhat lower than 1 keV is realistic for a microbulk readout in an experiment like TREX-DM. Indeed, the target threshold of the experiment in its baseline configuration is 0.4 keV. The huge physics potential of lowering this parameter (potentially down to the single-electron level, ~ 20 eV) in terms of improved sensitivity (see Section 4) has prompted the investigation to increase the operational gain of the readout, by means of a preamplification stage that multiplies the primary electron cloud before entering the microbulk gap, effectively contributing with an additional multiplication factor to the final readout gain. This preamplification stage consists of a Gas Electron Multiplication (GEM) foil, which is made of a copper-clad (on both sides), 50-µm-thick kapton foil, perforated by a high density, regular matrix of holes. The primary electrons go through the holes and get multiplied by a factor depending on the voltage applied between the electrodes. The raw materials of GEM foils are identical to the ones of microbulk planes, which makes this option promising for low-background searches.

In this article we report on a study of a readout combining microbulk plus GEM. Although the combination of a GEM and a Micromegas (GEM + MM henceforth) has been tested in the past ^17^, this is the first time this is done with a microbulk Micromegas, at high pressures, and in the context of low-background constraints. This is also, to our knowledge, the first time that a combined GEM + MM readout is seriously considered for installation in a real experiment.

In order to perform the study, a test set-up has been built and operated, as described in Subsection 2.1. The results of the characterization of the combined readout are presented in Subsection 3.1. Later on, another set-up with a full-scale GEM foil installed on top of an exact replica of the TREX-DM microbulks, simulating the real installation to be done in the TREX-DM experiment, is prepared to demonstrate the feasibility of this solution to enhance the threshold of the experiment. The description and results with this larger-scale set-up are presented in Subsection 2.2 and Subsection 3.2. We briefly discuss in Section 4 the sensitivity projections that the improvement in threshold of this work could potentially bring to an enhanced TREX-DM experiment. We finish with our conclusions in Section 5.

## 2 Methods

This section is devoted to the description of the experimental set-ups used to obtain the results presented in this article.

### 2.1 Description of the test set-up

The microbulk Micromegas detector with the GEM preamplification stage is placed inside a small (2.4 L) stainless-steel chamber certified to withstand 12 bar. The vacuum level achieved in this chamber after ~ 1 h with a Pfeiffer Vacuum HiCube 80 Classic Turbo Pump is ~ 10 ^*–*5^ mbar. The gas used in these studies is Ar-1%iC _4_H _10_, though it is intended to extend them to other Ar- and Ne-based mixtures of interest to TREX-DM (such as Ar-10%iC _4_H _10_).

The microbulk Micromegas detector lies on top of a metallic support plate, separated from it by a PTFE piece. The Micromegas used has a non-segmented and disc-shaped anode with a small 2-cm-diameter circular active area (cathode). The gap between mesh and anode is 50 µm. Several Micromegas with varying hole diameters (50–60 µm) and hole pitch (100–110 µm) are used in these tests. A GEM stage of roughly the same active area is mounted on top of the mesh, at a distance *L*_transfer_ = 10 mm. The GEM has a thickness of 60 µm (50 µm the Kapton, 5 µm each copper layer), hole pitch of 140 µm, diameter of holes in copper of 70 µm and diameter of holes in Kapton of 60 µm. Finally, a cathode (a stainless-steel grid) is placed above the GEM, at a distance *L*_drift_ = 13 mm. The cathode has a ^55^Fe source attached (K-alpha x-ray at 5.9 keV) facing the ionization volume.

As for the voltages, the anode is kept grounded, the mesh at *V*_mesh_, the bottom and top layers of the GEM at *V*_bottom_ and *V*_top_, respectively (we define the GEM preamplification voltage as *V*_GEM_ = *V*_top_ *– V*_bottom_), and the cathode at *V*_cath_. The metallic support plate is kept at *V*_plate_ = *V*_mesh_ in order to avoid potential distortions in the transfer field *E*_transfer_. Two CAEN HV power supply modules (a 4-channel N1471H and a 2-channel N471A) are used to provide these voltages.

**Figure 1.**
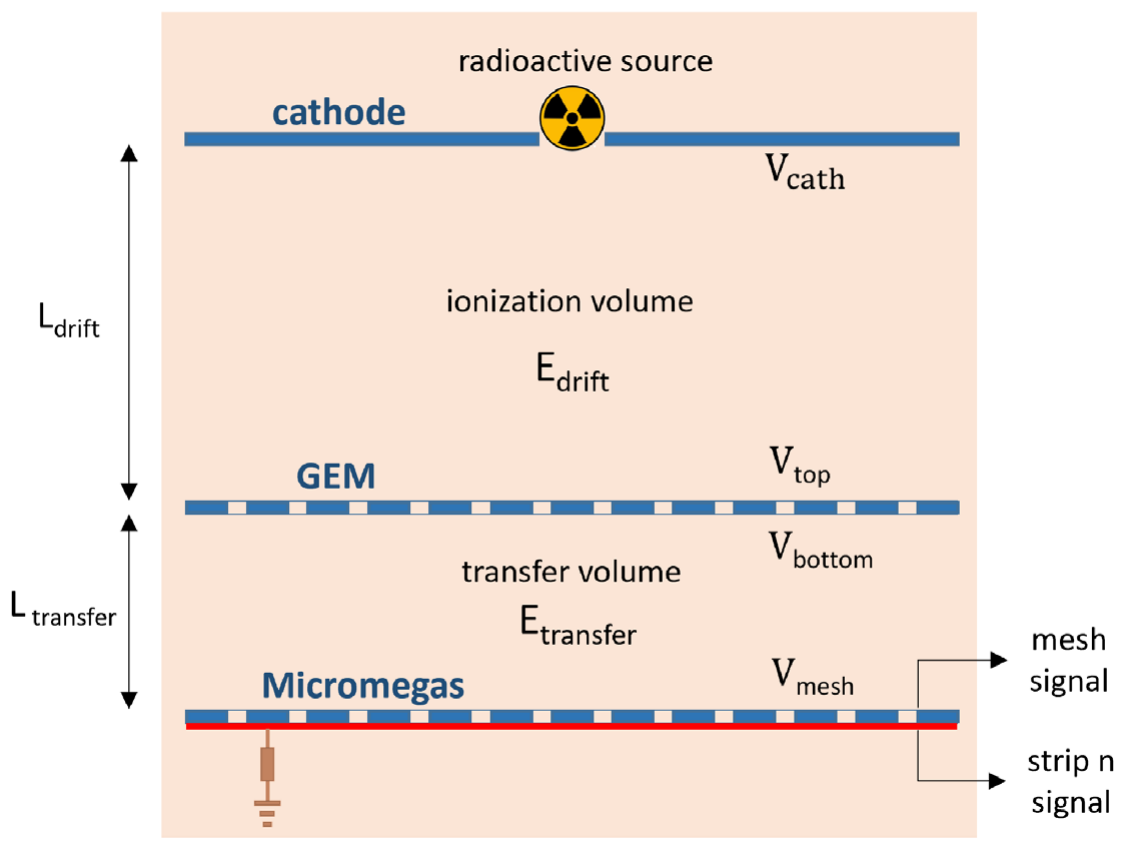
Schematic view of the different elements of the set-up (Micromegas, GEM, cathode, calibration source) along with the relevant parameters ( *L*_transfer_, *L*_drift_, *E*_transfer_, *E*_drift_, *V*_mesh_, *V*_bottom_, *V*_top_, *V*_cath_).

**Figure 2.**
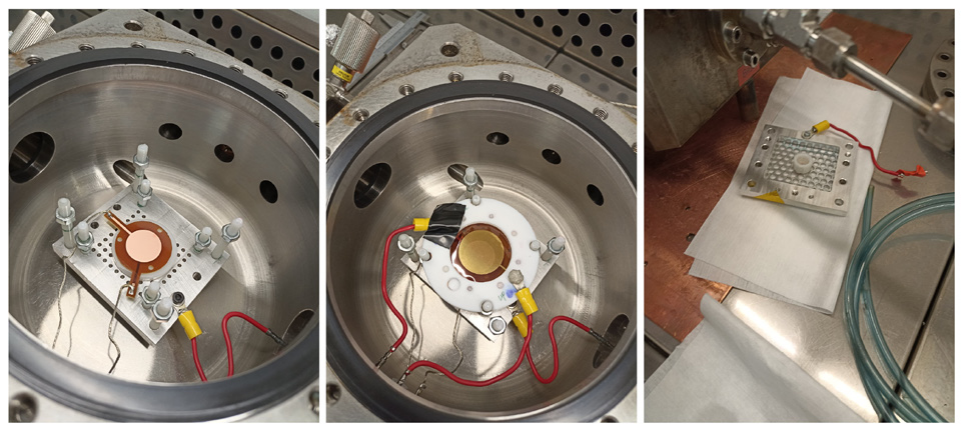
Left: metallic support plate inside the vessel with the 2-cm-diameter microbulk Micromegas on top. The inner PTFE pillars support the GEM at the appropriate distance, while the outer PTFE pillars are used to hold the cathode. The anode, mesh and plate connections to the feedthroughs are shown. Center: GEM foil mounted on top of the Micromegas, along with the HV connections. Right: Cathode with ^55^Fe source attached facing down.

A schematic view of the set-up can be seen in Figure 1, and images of the different elements are shown in Figure 2.

Regarding the DAQ, the signal from the anode is first sent to a preamplifier (Canberra Model 2005), and then it goes through an amplifier module (Canberra Model 2022 NIM module). Both the preamplified and the amplified signals are read with an oscilloscope (a Tektronix TDS5054). A custom-made data-taking and analysis software is used to control the oscilloscope and process the data.

### 2.2 Description of the full-scale TREX-DM set-up

As already explained in Section 1, the motivation to explore the combination GEM + MM detector is to reduce the energy threshold in the low-mass WIMPs search carried out by TREX-DM. Therefore, although the results for a small set-up that will be described in Subsection 3.1 look promising, a deeper investigation is required to determine if they are also achievable in real experimental conditions (essentially, a much larger readout area and drift distance). To this end, a test bench is prepared with a stainless-steel 50 L chamber containing a spare detector identical to the ones installed in TREX-DM and a GEM foil on top. This chamber achieves a vacuum level of ~ 10 mbar using a Pfeiffer Vacuum HiCube 80 Classic Turbo Pump during ~ 1 h, then it is filled with Ar-1%iC _4_H _10_, and a flow of 8 l h ^-1^ is set during 72 h in order to ensure good quality of the gas. During these tests, the pressure has been set to 1 bar due to design specifications of the chamber. The microbulk Micromegas detector lies on top of the endcap of the chamber. As already mentioned, the readout plane has a 25×25 cm ^2^ square active area, patterned with 512 strips (256 en each direction) with the mesh gap being 50 µm. A GEM foil of the same dimensions and gap is placed above the mesh, at a distance *L*_transfer_ = 10 mm. Finally, the cathode (a stainless-steel grid) is placed above the GEM, at a distance *L*_drift_ = 100 mm. The cathode has two ^109^Cd radioactive sources attached (K-alpha x-ray at 22.1 keV).

Again, as described in Subsection 2.1, the mesh is kept at *V*_mesh_, the bottom and top layers of the GEM at *V*_bottom_ and *V*_top_, with *V*_GEM_ = *V*_top_ *– V*_bottom_, and the cathode at *V*_cath_. The same CAEN HV power supply N1471H is used in this test. To read out the signals from the TPC, a combination composed of a Front-End Card (FEC) with AGET (ASIC for General Electronic readout of TPCs) chips ^18^ and a Feminos card ^19^ is used. Both the FEC and the Feminos are custom-made electronics cards developed by CEA Saclay as a solution for data acquisition in nuclear and high-energy physics experiments. Each FEC has 4 AGETs, each of them with 64 channels, which makes them ideal for high-granularity readouts such as the ones used in TREX-DM. The AGETs generate the trigger based on the signal (it can be fine-tuned to trigger on single-channel pulses), and they provide the amplification, shaping and storage of the analog signals. On the other hand, the Feminos interfaces with the AGETs to digitize the analog signals (with 12-bit precision) and aggregate them into coherent events. This allows for high-speed data transfer from the Feminos towards the back-end DAQ, namely a computer using a custom-made software to interface with the Feminos card and handle the data acquisition and storage into .aqs files (raw data files). To process and analyze the data, an analysis routine based on REST-for-Physics ^20^, a custom-made software framework developed for the analysis of data from rare event search experiments, is implemented.

**Figure 3.**
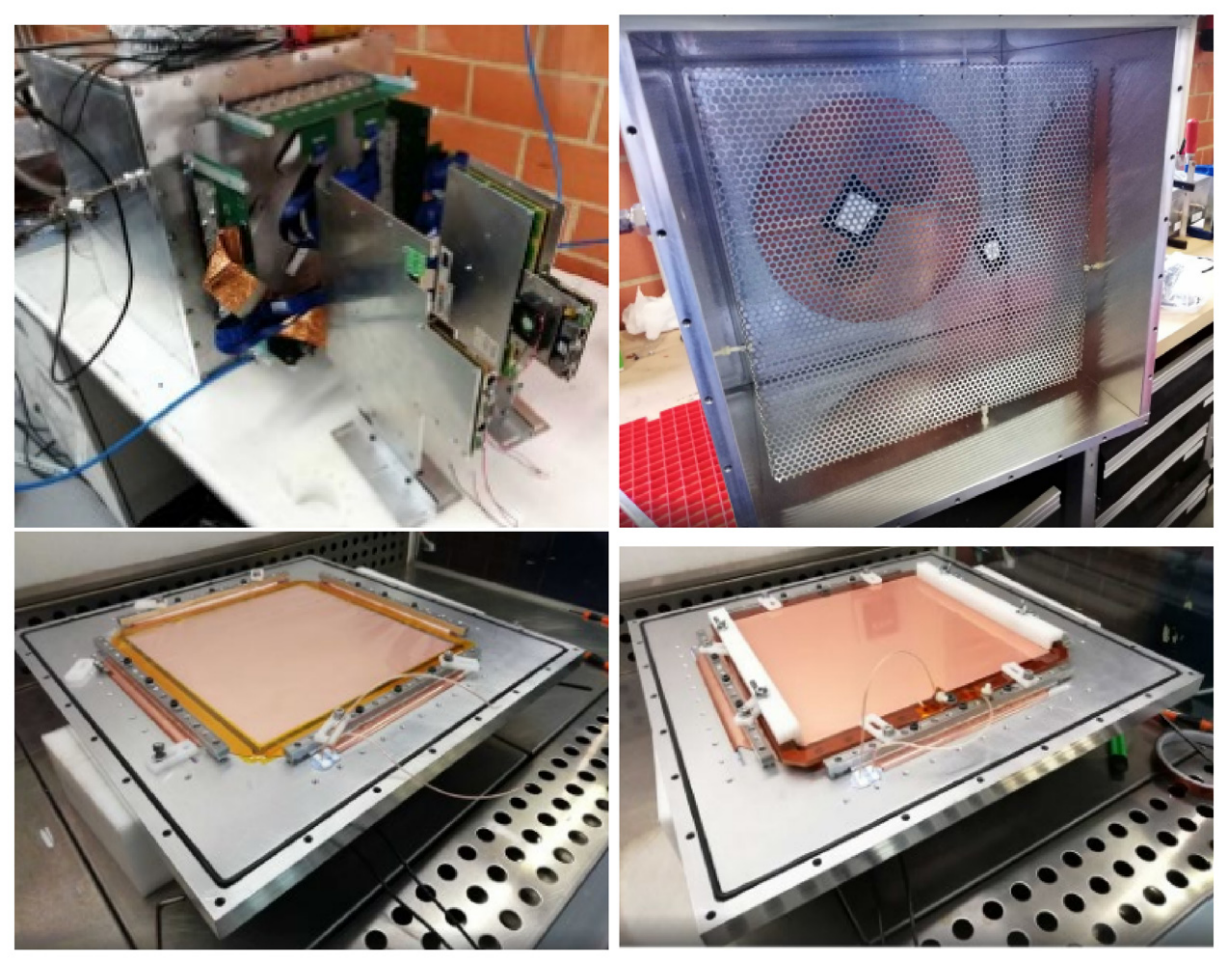
Top left: closed vessel with DAQ. Top right: cathode with two ^109^Cd radioactive sources (attached with black tape). Bottom left: Micromegas detector secured on the endcap of the chamber. Bottom right: GEM foil placed on top of the mesh.

The schematic view of the set-up is shown in Figure 1, and images of the assembly of all the parts of the set-up can be seen in Figure 3.

## 3 Results

This section contains the main results derived from the operation of the test set-up and the full-scale setup described in Section 2.

### 3.1 Results from the test set-up

The goal is to obtain the relative amplification factor provided by the extra GEM stage with respect to only-Micromegas runs. To this end, both only-Micromegas ( *V*_mesh_ ON, *V*_GEM_ = 0) and Micromegas+GEM ( *V*_mesh_ and *V*_GEM_ ON) calibration runs are taken. We define the preamplification factor as the extra gain added by the GEM with respect to a fixed Micromegas-induced gain. This is the natural extra gain parameter that arises when adding a second amplifying stage. However, in real experimental conditions, the maximum voltage that can be reached with the Micromegas alone is higher than the one that can be achieved with the GEM stage. Therefore, we define the GEM effective extra gain factor, GEM extra factor for short, as the amplification provided by the GEM in the optimized GEM + MM set-up with respect to the optimized only-Micromegas set-up. The chosen gas mixture for this study is Ar-1%iC _4_H _10_, due to its importance both for IAXO ^21^ and TREX-DM, the main experimental pursuits of the authors, and its immediate availability. Both the preamplification factor and the GEM extra factor are obtained at 1, 4 and 10 bar (target pressure in TREX-DM). The only missing value is the preamplification factor at 1 bar, due to noise problems in the set-up at that pressure the day when those data were taken. However, since the relevant parameter and focus of our efforts, the GEM extra factor, had already been measured, we proceeded with the rest of the measurements.

**Figure 4.**
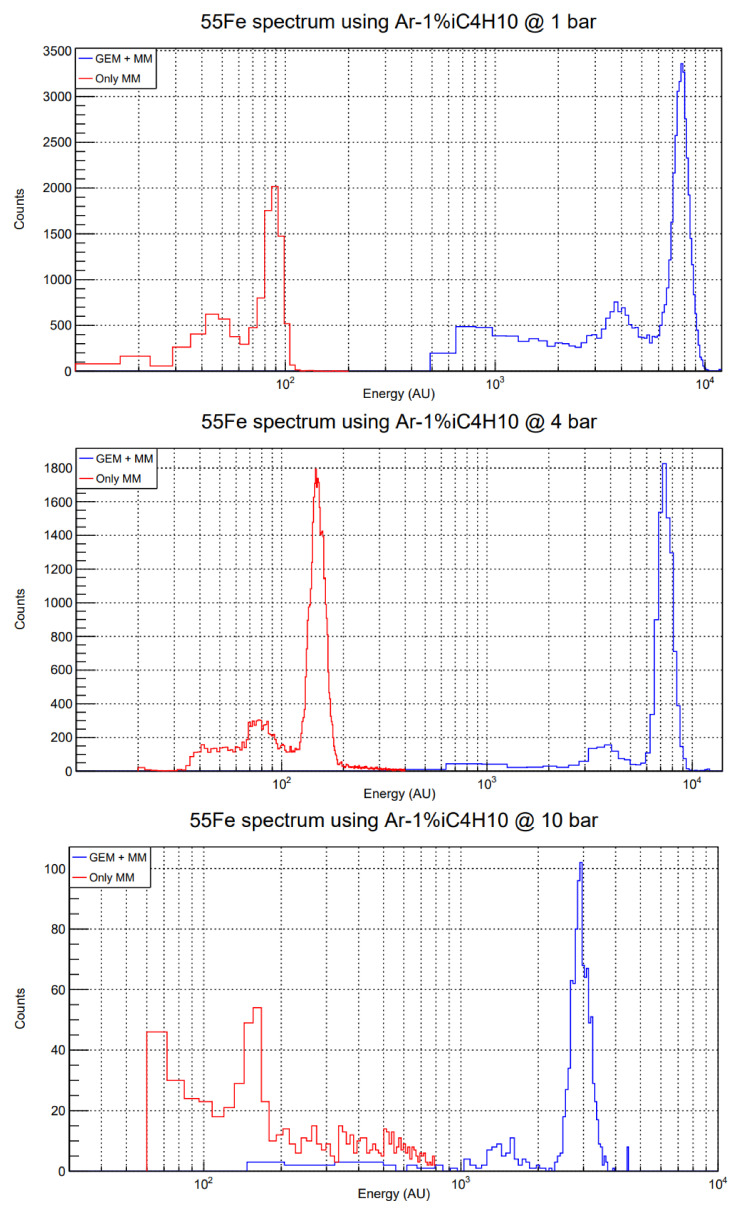
Energy spectra comparison between only MM (red) and GEM + MM (blue) calibrations using a ^55^Fe source in the test set-up. The gas mixture is Ar-1%iC _4_H _10_. Note that the horizontal axis is presented in logarithmic scale. Top: 1 bar, GEM extra factor ≈ 90; middle: 4 bar, GEM extra factor ≈ 50; bottom: 10 bar, GEM extra factor ≈ 20. The voltages of these runs are the ones recorded in the fifth column (red lines) and second and third columns (blue lines) of Table 1.

The comparison of both GEM + MM and only-Micromegas spectra is done within the same dynamical range of a given electronics setup, to avoid systematics derived from different electronic gains. Thus, one must be careful selecting the parameters of the DAQ electronics, as it is easy to saturate the amplifier module with the GEM-preamplified signals. In this way, direct comparison of energy spectra like the ones shown in Figure 4 can be made.

The operation points with highest stable voltages achieved for the set-up described in Subsection 2.1 are summarised in Table 1. A reference value for the maximum voltage for only *V*_mesh_ runs was obtained from 22. For combined runs, the starting point for both *V*_mesh_ and *V*_GEM_ was a safe value, around 30–40 V below the reference voltage. From that value, the voltage was raised little by little, in 5 V increments, first in *V*_mesh_ and then in *V*_GEM_, until reaching unstable behaviour (generally sparks). It was observed, however, that for the last stable voltage in *V*_mesh_, *V*_GEM_ could still be pushed a bit further up.

**Table 1.** GEM extra factors and preamplification factors achieved for the two set-ups at different pressures, defined as the gain ratio between GEM + MM runs ( *V*_GEM_ ≠ 0 V) and only Micromegas runs ( *V*_GEM_ = 0 V). The first three entries correspond to the test set-up, while the fourth line belongs to the full-scale set-up.

Pressure (bar)	*V*_mesh_ (V) (GEM + MM system)	*V*_GEM_ (V) (GEM + MM system)	Preamp. factor	*V*_mesh_ (V) (only-MM system)	GEM effective extra gain factor
1	305	310	-	315	90
4	390	410	70	400	50
10	535	550	21	540	19
1	290	285	85	293	80

Given that the purpose of this set-up was just to prove the feasibility and potential of the combined GEM + MM system when installed in real experimental conditions in TREX-DM, the in-depth study about the electron transmission (and gain curves) of the GEM + MM was left for the full-scale set-up in Subsection 3.2. Here, in all cases, *E*_drift_ = 100 V cm ^-1^ bar ^-1^, *E*_transfer_ = 100 V cm ^-1^ bar ^-1^, values which are usually within the electron transmission plateau of the Micromegas. However, border effects cannot be excluded given the size of the active areas and the lack of a field shaper.

Comparison of the position of the 5.9 keV peak in ^55^Fe calibration runs points to maximum GEM extra factors of 90 (1 bar), 50 (4 bar) and 20 (10 bar). These factors have been reproduced over several days and for different Micromegas detectors with the same micropattern, and all of them are contained within a range of *±*20%. This difference is mainly due to slight variations in the highest stable voltages achieved (usually *±* 5 V either in *V*_mesh_, *V*_GEM_ or both). The decrease in gain with pressure is expected in MPGDs in general (even though microbulk Micromegas do not display such a pronounced performance degradation at high pressures) ^23^.

Several examples of this comparison are shown in Figure 4. In the case of 10 bar, the only-Micromegas runs are more difficult to take, because the mean free path of 5.9 keV photons in Ar-1%iC _4_H _10_ at that pressure is 2.3 mm ^24^, and those that do not get absorbed in the drift volume have to go through the GEM foil. Therefore, the exponential background is already noticeable and the calibration peak is less intense, but still clearly visible. All the resolutions (in %FWHM), with and without preamplification, are around 20%, except for the only-Micromegas run at 10 bar, which is around 30%, mainly due to the problem with the small number of events mentioned above. However, this result points to no significant degradation in resolution when adding a preamplification stage.

### 3.2 Results from the full-scale TREX-DM set-up

Although the initial goal was to replicate the results from the small set-up at 1 bar discussed in Subsection 3.1, some more tests are performed in this full-scale set-up.

**Figure 5.**
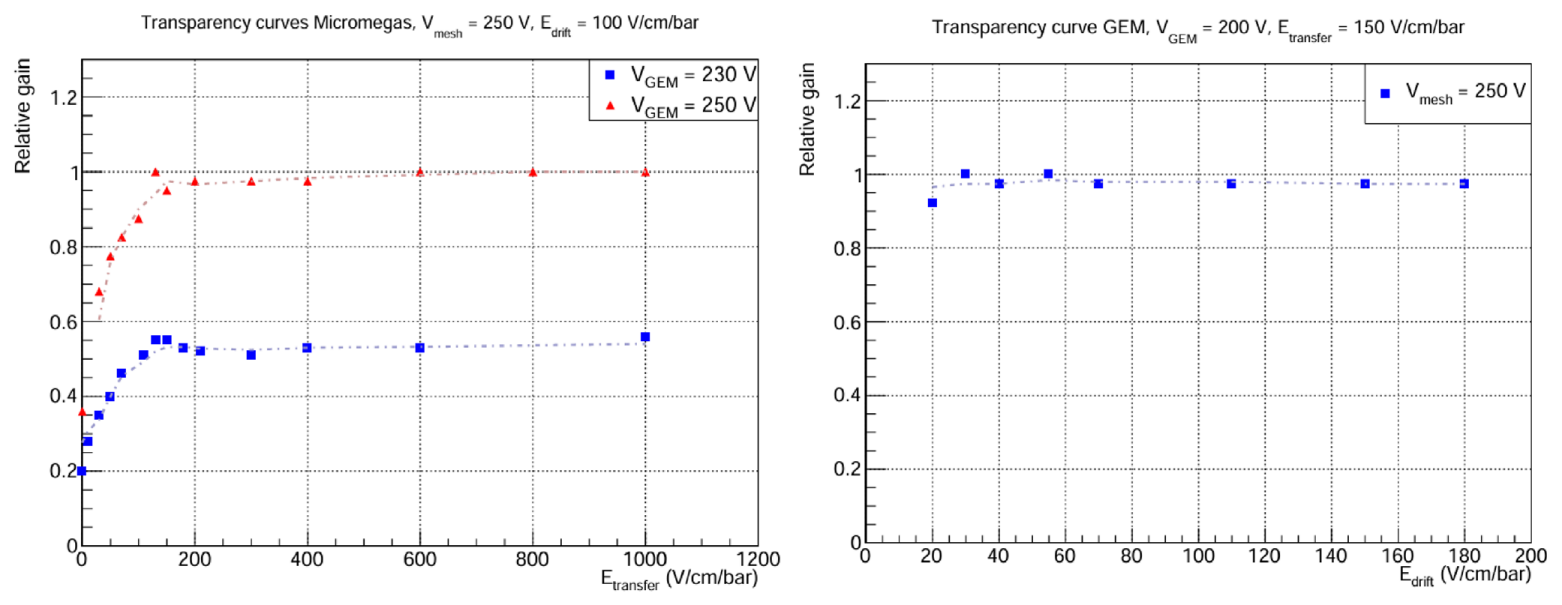
Electron transmission curves. The *y* axis corresponds to the mean peak position, normalized to the highest value. Statistical errors in both plots are < 1%. Left: Micromegas transmission for fixed mesh voltage and two different GEM voltages. *E*_drift_ = 100 V cm ^-1^ bar ^-1^ because there is total transparency in the GEM for that drift field. Right: GEM transmission for fixed mesh and GEM voltages. *E*_transfer_ = 150 V cm ^-1^ bar ^-1^ in order to be at the plateau of the Micromegas transparency curve.

In particular, the electron transmission (transparency curves) of the GEM foil and the mesh is studied. The results are shown in Figure 5. As for the GEM transmission, it can be seen that a plateau is reached very quickly, even for very low drift field values. This is expected, because in previous studies of electron transmission in GEMs ^25^, it has been shown that collection efficiency increases with *V*_GEM_, meaning that full transparency is achieved with lower *E*_drift_ as *V*_GEM_ goes up. Regarding the Micromegas curves, an interesting phenomenon occurs at *E*_transfer_ = 0 V cm ^-1^ bar ^-1^, because the relative gain has a non-zero value: the photons converted in the drift volume are amplified through the GEM, and thanks to the proximity to the mesh, diffusion is enough for some of the events to reach the Micromegas, where they are amplified again. This effect is possibly explained by the fact that *L*_transfer_ is small, ~ 1 cm, but tests at different transfer distances would be necessary to shed light on this. On the other hand, the relative gain remains roughly constant from *E*_transfer_ ≈ 150 V cm ^-1^ bar ^-1^. This result is unanticipated, because the transparency curve is expected to depend on the extraction efficiency of the GEM bottom layer and the collection efficiency of the Micromegas. While this plateau is usual for the Micromegas collection efficiency, one would expect the extraction efficiency of the GEM to increase in this region, up to a few kV cm ^-1^ bar ^-1^ ^26^. We do not have a conclusive explanation for this, but several hypothesis are being considered:

- What we think is a plateau is a slowly rising curve. Due to voltage limitations of the set-up, it is not possible to go further beyond ~ 1 kV cm ^-1^ bar ^-1^, so perhaps there is a gain increase up to a few kV cm ^-1^ bar ^-1^.
- The gas mixture is playing an important role in the shape of the curves. Normally, GEM detectors use noble gases in combination with gases such as CH ^4^ or CF ^4^ C ^4^H ^10^ due to their high drift velocities and low diffusion coefficients with respect to other quenchers such as iC _4_H _10_^27^. To the best of our knowledge, the mixture Ar-1%iC _4_H _10_ has not been characterised in the context of GEMs, and it could be the case that its higher diffusion coefficients imply a loss of extraction efficiency from the bottom layer of the GEM.

Irrespective of the reason, it should be noted that this unexplained behaviour does not invalidate the results: at most, an optimisation of the extraction efficiency would yield higher gains, and thus larger GEM extra factors.

**Figure 6.**
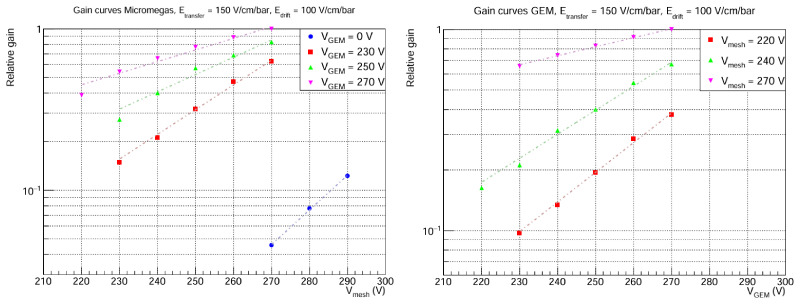
Gain curves. The *y* axis corresponds to the mean peak position, normalized to the highest value. Statistical errors in both plots are < 1%. Left: Micromegas curves for fixed *V*_GEM_ . Right: GEM curves for fixed *V*_mesh_. In both cases, *E*_drift_ and *E*_transfer_ are fixed. Note that the maximum gain corresponds to the same data point in both plots ( *V*_mesh_ = 270 V, *V*_GEM_ = 270 V), so the relative gain is directly comparable between plots.

Also, gain curves are studied before the determination of the maximum GEM extra factor. As it can be seen in Figure 6, two types of gain curves are examined: Micromegas gain curves (varying *V*_mesh_ for a fixed *V*_GEM_ ) and GEM gain curves (varying *V*_GEM_ for a fixed *V*_mesh_). In both cases, the expected exponential behaviour with amplification voltage is observed. In the Micromegas curves, the special case *V*_GEM_ = 0 V is also included, which corresponds to the baseline only-Micromegas detector. Comparison of this curve with the GEM + MM curves already hints at extra gain factors of O(10) thanks to the GEM addition. In all cases, it can be seen that the curves are not perfectly parallel (in log scale). This suggests both stages are not totally factorizable, maybe due to some backflow. We do not have a sound explanation for this and it should be explored further, but again it is not important for the purpose of this paper.

**Figure 7.**
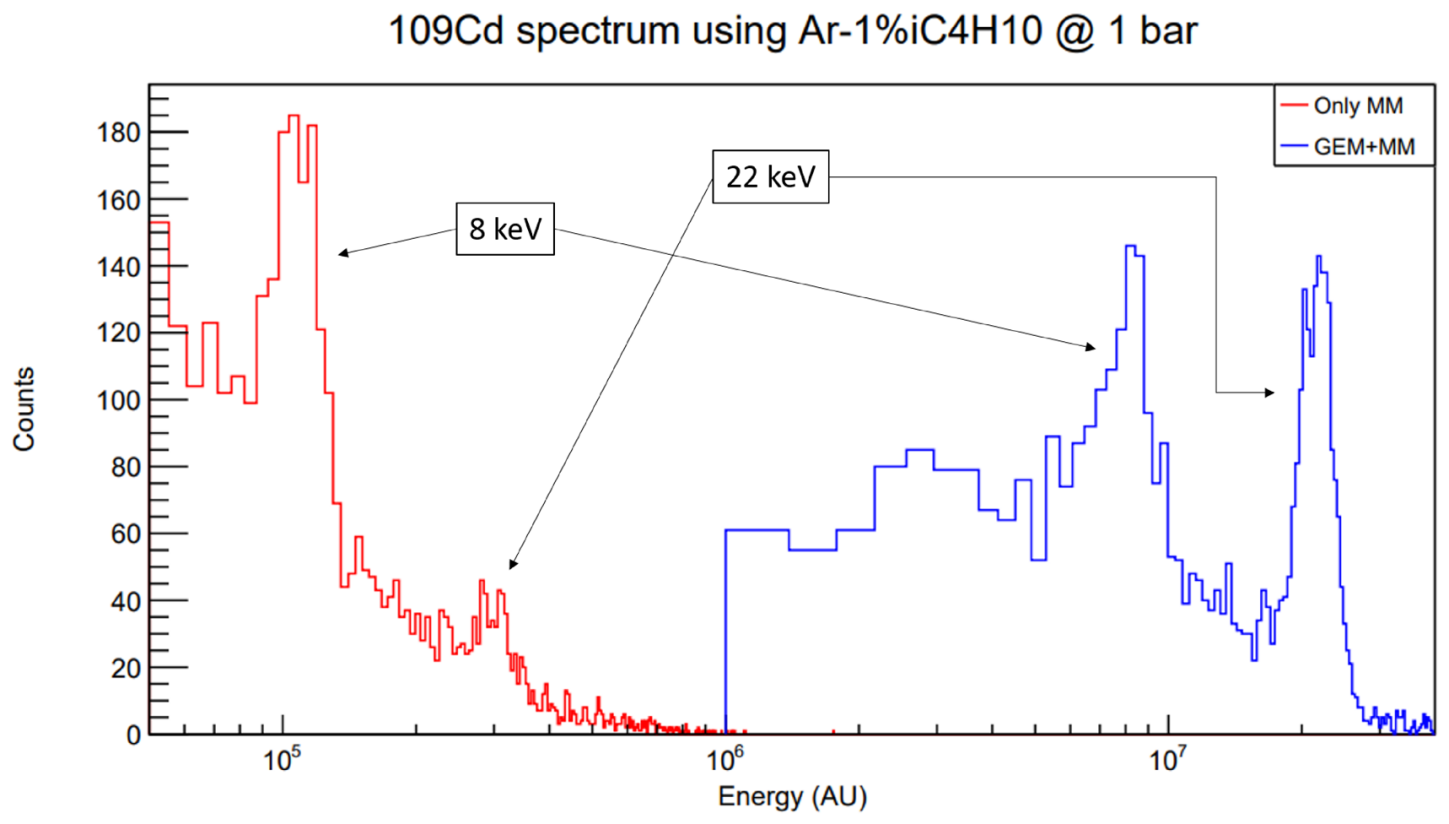
Energy spectrum comparison between only MM and GEM + MM calibrations using a ^109^Cd source in the full-scale set-up. The 8 keV peak corresponds to the copper fluorescence at the Micromegas surface. The gas mixture is Ar-1%iC _4_H _10_ at 1 bar. Note the horizontal axis is presented in logarithmic scale, and that an energy cut has been applied to the GEM + MM run in order to remove background and keep the left part of the canvas clean.

Lastly, some more data are taken in order to explore the value for the maximum GEM extra factor and preamplification factor achievable. In the fourth row of Table 1, the achieved voltages are shown. Comparison of the position of the 8 keV copper fluorescence peak in ^109^Cd calibration runs (see Figure 7) yields a GEM extra factor of 80, in line with the result obtained in Subsection 3.1. In these runs, *E*_drift_ = 100 V cm ^-1^ bar ^-1^, *E*_transfer_ = 150 V cm ^-1^ bar ^-1^ as suggested by the transparency curves.

Note that the voltages are lower than those presented in Subsection 3.1 because of the intrinsic difficulty associated to operating larger-area Micromegas (1 vs. 1024 channels means higher possibility of leakage currents between mesh and some channels). Despite that, the potential to lower the energy threshold is present even in the full-scale set-up, mimicking the real experimental conditions of TREX-DM.

## 4 Discussion

This section discusses the impact the results presented in Section 3 have on the sensitivity of TREX-DM. As argued in the introduction, there is a strong motivation to extend the sensitivity of dark matter experiments to lower WIMP masses. This requires lowering energy thresholds, as well as reducing the background at the lowest energies.

As of 2022, TREX-DM achieved a background level at low energies of around 80 dru (dru = c keV ^-1^ day ^-1^ kg ^-1^), and an energy threshold around 900 eV _*ee*_. Reducing the threshold from 900 eV _*ee*_ to 50 eV _*ee*_ significantly enhances sensitivity in the < 1 GeV c ^-2^ region. As already demonstrated in this paper, a great improvement in energy threshold can be achieved by introducing a new electron preamplification stage (the GEM) atop the Micromegas. Laboratory tests ( Subsection 3.1 and Subsection 3.2) have proven GEM extra factors ranging from 20 to 90 are feasible, contingent on gas pressure.

**Figure 8.**
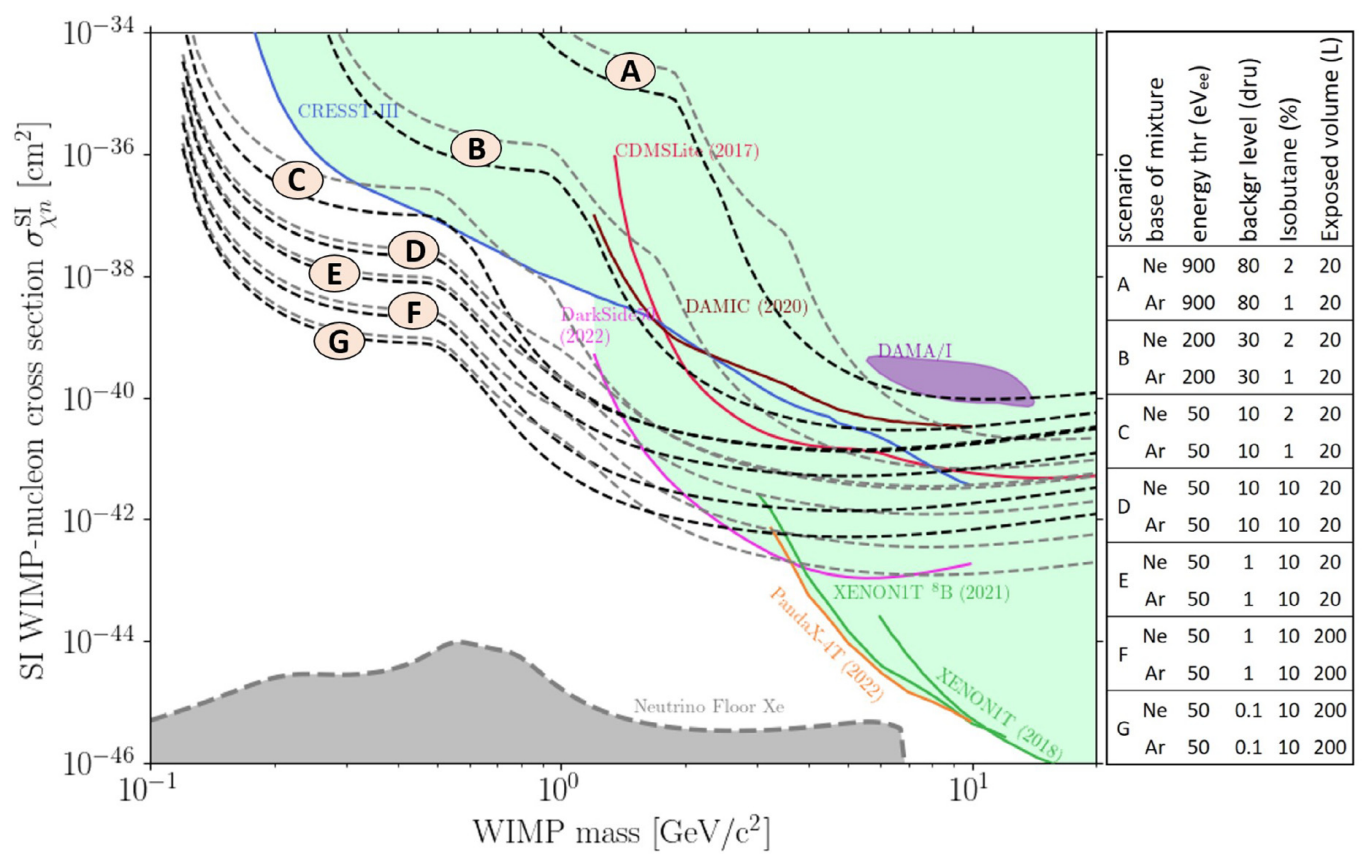
WIMP-nucleon cross-section vs. WIMP mass exclusion plot, with current bounds from experiments ^28– 34^, claimed discovery ^35^ and different scenarios for TREX-DM (all of them 1 year of exposure time). Each scenario is plotted with Ne-based (black) and Ar-based (grey) mixtures. Also, solar neutrino floor with a Xe target is shown ^36^.

In Figure 8, the sensitivity projections in TREX-DM for Spin Independent WIMP-nucleon interaction over a year are shown, considering several experimental parameters: energy threshold, background level and isobutane content of the gas mixture. The background level is expected to improve in the near term (there is a roadmap of upgrades underway, but the details are beyond the scope of this paper). On the other hand, optimizing the gas mixture plays a pivotal role. Sensitivity estimates indicate better performance with neon-based mixtures compared to argon. Also, argon mixtures with increased isobutane content are being considered: more isobutane enhances sensitivity to WIMPs below 1 GeV c ^-2^ due to the lower mass of target nuclei. Even though these mixtures have not been studied in this paper, the preamplification results shown here are expected to hold because Ar-1%iC _4_H _10_ is a conservative mixture choice: neon-based mixtures typically provide higher gains ^22^, and an increased isobutane content also goes in the direction of attaining greater amplifications.

## 5 Conclusions

Electron amplification in gas offers an attractive strategy to increase signal to noise ratio and therefore to reduce detector energy thresholds. This feature, coupled with the ability of building radiopure readout Micromegas planes with the microbulk technology, is at the core of the TREX-DM proposal. In this paper we have demonstrated the feasibility of an amplification scheme that should allow to approach the single-electron sensitivity in realistic TREX-DM implementations. The addition of a GEM preamplification stage on top of the microbulk readout, allows for this gain, without jeopardizing the radiopurity specifications of the readout. At the time of writing this article, a GEM + MM combined readout like the one tested in Subsection 2.2 is being installed and commissioned at the TREX-DM experiment. As discussed in Section 4, this improvement would open a new detection window at lower recoil energies that, depending on the background levels achieved at those energies, might lead to substantial improvement to low mass WIMPs, potentially down to unexplored region of the parameter space.

## Data Availability

Zenodo: Data for manuscript submitted to Open Research Europe with title "Micromegas with GEM preamplification for enhanced energy threshold in low-background gaseous time projection chambers", https://doi.org/10.5281/zenodo.14525554 This project contains the following underlying data: Spectra for the test set-up: spectrum_test_set-up_1_bar_only_MM.root (1 bar, only MM) spectrum_test_set-up_1_bar_GEM_MM.root (1 bar, GEM+MM) spectrum_test_set-up_4_bar_only_MM.root (4 bar, only MM) spectrum_test_set-up_4_bar_GEM_MM.root (4 bar, GEM+MM) spectrum_test_set-up_10_bar_only_MM.root (10 bar, only MM) spectrum_test_set-up_10_bar_GEM_MM.root (10 bar, GEM+MM) Spectrum for the full-scale set-up: spectrum_full-scale_set-up_1_bar_only_MM.root (1 bar, only MM) spectrum_full-scale_set-up_1_bar_GEM_MM.root (1 bar, GEM+MM) Transparency curves for the full-scale set-up: transparency_curve_micromegas_Edrift100_Vmesh250_Vgem230_full-scale_set-up.txt transparency_curve_micromegas_Edrift100_Vmesh250_Vgem250_full-scale_set-up.txt transparency_curve_gem_Etransfer150_Vmesh250_Vgem200_full-scale_set-up.txt GEM gain curves for the full-scale set-up: gain_curve_gem_Etransfer150_Edrift100_Vmesh220_full-scale_set-up.txt gain_curve_gem_Etransfer150_Edrift100_Vmesh240_full-scale_set-up.txt gain_curve_gem_Etransfer150_Edrift100_Vmesh270_full-scale_set-up.txt Micromegas gain curves for the full-scale set-up: gain_curve_micromegas_Etransfer150_Edrift100_Vgem0_full-scale_set-up.txt gain_curve_micromegas_Etransfer150_Edrift100_Vgem230_full-scale_set-up.txt gain_curve_micromegas_Etransfer150_Edrift100_Vgem250_full-scale_set-up.txt gain_curve_micromegas_Etransfer150_Edrift100_Vgem270_full-scale_set-up.txt Note: the .root format is associated to ROOT, the CERN open-source data analysis framework ( https://root.cern/). Data are available under the Creative Commons Attribution 4.0 International license (CC-BY 4.0) https://creativecommons.org/licenses/by/4.0/
