# Supplementary material for: Micromegas with GEM preamplification for enhanced energy threshold in low-background gaseous time projection chambers
Source: Open Res Eur. 2025 Mar 18;5:53. Originally published 2025 Feb 18. [Version 2] doi: 10.12688/openreseurope.19258.2 (PMC11926529; doi:10.12688/openreseurope.19258.2)
Supplement: Peer review history

# Reviewer report: Reviewer response for version 2

**DOI:** 10.21956/openreseurope.21540.r52294

**Published:** 2025-04-02

**Authors:** Ludovic Mathieu

**Affiliations:** 1 Laboratoire de Physique des 2 infinis, Orsay, France

This paper presents an innovative approach to boost the gain of gaseous detector by combining GEM and Micromegas technology: primary electrons are multiplied by the GEM planed before being multiplied again in the Micromegas stage. This structure has several additional parameters (GEM spacing, GEM voltage, drift field) compared to Micromegas-only detectors, the influence of which are studied in this paper.

 The aim of the gain improvement is to lower the energy threshold of such detector. This will be of primary importance for dark matter detection. This paper presents experiments carried out on a test setup-up, as well as its transposition to the full-scale TREX-DM setup.

 Several comments have already been done by the two previous reviewers, and they have been addressed by the authors.

 I don’t have major corrections to request, just minor remarks:

- section 3.1, concerning the variation between different Micromegas detectors: the mesh is placed above the detection plane on pillars ; even if checked the fabrication process can induce small variations of the pillars height, inducing non-negligible differences on the Micromegas gain
- gain: the authors explain the gain increase they measure, but never mention the absolute value of this gain ; although not mandatory for their results, this information could be an interesting addition
- maximum voltage: the authors announce that the maximum voltage is lower for GEM+MM than it is for MM-only ; an explanation (or hypothesis) could be valuable

Best regards

Is the study design appropriate and does the work have academic merit?

Yes

Is the work clearly and accurately presented and does it cite the current literature?

Yes

If applicable, is the statistical analysis and its interpretation appropriate?

Not applicable

Are all the source data underlying the results available to ensure full reproducibility?

Yes

Are the conclusions drawn adequately supported by the results?

Yes

Are sufficient details of methods and analysis provided to allow replication by others?

Yes

Reviewer Expertise:

Nuclear instrumentation

I confirm that I have read this submission and believe that I have an appropriate level of expertise to confirm that it is of an acceptable scientific standard.

# Reviewer report: Reviewer response for version 2

**DOI:** 10.21956/openreseurope.21540.r52247

**Published:** 2025-03-20

**Authors:** Ignacio Lázaro Roche

**Affiliations:** 1 Avignon University, Rustrel, France; 2 CNRS Delegation Provence et Corse, CNRS Delegation Provence et Corse, Rustrel, Provence-Alpes-Côte d'Azur, France

The version 2 of the article takes into account all the remarks made by the 2 reviewers and is suitable for acceptation.

Is the study design appropriate and does the work have academic merit?

Yes

Is the work clearly and accurately presented and does it cite the current literature?

Partly

If applicable, is the statistical analysis and its interpretation appropriate?

I cannot comment. A qualified statistician is required.

Are all the source data underlying the results available to ensure full reproducibility?

Partly

Are the conclusions drawn adequately supported by the results?

Yes

Are sufficient details of methods and analysis provided to allow replication by others?

Partly

Reviewer Expertise:

-MPGD detectors, micromegas, time projection chambers, underground science, low background, dark matter

I confirm that I have read this submission and believe that I have an appropriate level of expertise to confirm that it is of an acceptable scientific standard.

# Reviewer report: Reviewer response for version 2

**DOI:** 10.21956/openreseurope.21540.r52248

**Published:** 2025-03-19

**Authors:** David Attié

**Affiliations:** 1 Université Paris-Saclay, Gif-Sur-Yvette, France

Dear Authors

 Most of the corrections and comments have been considered.

 Only one minor correction is missing:

 - §3.1

     > In 5th paragraph, change "microbulk" to "Microbulk".

 After this very minor correction, the article can be indexed.

 Best regards,

 The reviewer.

Is the study design appropriate and does the work have academic merit?

Yes

Is the work clearly and accurately presented and does it cite the current literature?

Partly

If applicable, is the statistical analysis and its interpretation appropriate?

Not applicable

Are all the source data underlying the results available to ensure full reproducibility?

Yes

Are the conclusions drawn adequately supported by the results?

Yes

Are sufficient details of methods and analysis provided to allow replication by others?

Yes

Reviewer Expertise:

Micromegas detector, TPC, MPGD

I confirm that I have read this submission and believe that I have an appropriate level of expertise to confirm that it is of an acceptable scientific standard.

# Response

**Published:** 2025-03-20

**Authors:** Óscar Pérez Lázaro

**Affiliations:** Theoretical Physics, University of Zaragoza Department of Theoretical Physics, Zaragoza, Aragon, Spain

Dear reviewer, The minor typo we missed has been corrected. Thank you again for your comments and the thorough review of the paper. Best regards, The authors

# Reviewer report: Reviewer response for version 1

**DOI:** 10.21956/openreseurope.20846.r51384

**Published:** 2025-03-05

**Authors:** Ignacio Lázaro Roche

**Affiliations:** 1 Avignon University, Rustrel, France; 2 CNRS Delegation Provence et Corse, CNRS Delegation Provence et Corse, Rustrel, Provence-Alpes-Côte d'Azur, France

This article presents a study on the implementation of a preamplification stage using Gas Electron Multipliers (GEM) combined with microbulk Micromegas in gaseous time projection chambers (TPCs), aiming to lower the detection threshold. Two experimental setups are described: a small-scale test system and a larger-scale version with characteristics representative of TREX-DM.

 Section 2 provides a thorough technical description of the detectors and their operation, detailing key parameters such as system geometry, gas conditions, and electronic characteristics. The motivation behind this approach is well-justified, as reducing the detection threshold is critical for improving sensitivity to low-energy nuclear recoils. The authors highlight the potential of the GEM + Micromegas combination, particularly in terms of achieving higher effective gains and maintaining stable operation under experimental constraints.

 Section 3 provides a well-structured and data-rich presentation of GEM + MM performance. The results are encouraging for TREX-DM, particularly in achieving a lower energy threshold. However, the section would benefit from a more rigorous discussion of systematic uncertainties, theoretical interpretations, and potential improvements to the experimental design. Addressing these points would enhance confidence in the broader applicability of these findings to future large-scale detectors.

 While the manuscript is technically sound and the experimental methodology is well outlined, there are areas where further clarification would strengthen the study. Firstly, although previous works on GEM + Micromegas setups are mentioned, a more detailed discussion of how this implementation differs from or improves upon prior efforts would provide better context. Secondly, while the feasibility of the approach is demonstrated, the long-term stability of the combined readout under real experimental conditions (e.g., extended operation in TREX-DM) is not fully addressed. Finally, the potential impact on background levels due to the introduction of the GEM stage should be discussed in greater depth, as this is a critical factor in low-background experiments.

 Overall, this work presents a promising development in Micromegas-based TPCs for rare event searches, and the proposed approach could contribute to improved sensitivity in WIMP detection. However, additional discussion on long-term stability and potential drawbacks would enhance the completeness of the study.

**Strengths:**

 1. **Clear Experimental Design:** The distinction between the preamplification factor and the GEM extra factor is well-motivated, addressing the practical limitations of voltage stability in a GEM-MM configuration.

 2. **Comprehensive Data Acquisition:** Measurements at various pressures provide valuable insights into the scalability and practical performance of the setup under different experimental conditions.

 3. **Application-Oriented Approach:** The study is clearly aimed at improving the TREX-DM experiment’s sensitivity, with a focus on reducing the energy threshold, which aligns with the broader goal of enhancing low-mass WIMP detection.

**Areas for Improvement:**

 1. **Target Audience:** The article appears to be primarily aimed at the astrophysics community. However, addressing a broader audience by including other Micromegas + TPC user communities, such as the muography community, would be beneficial. This could be mentioned in the first paragraph of Section 1, along with references to the use of Micromegas in geophysics, civil engineering, and archaeology.

 2. **Limited Discussion on Systematic Errors:** While the results are presented with statistical confidence, there is little discussion on potential systematic uncertainties. For example, slight variations in voltage stability or differences in gas mixture purity could significantly impact gain factors.

 3. **Insufficient Justification for Missing Data:** The absence of a preamplification factor at 1 bar due to noise issues is noted, but further elaboration on the specific noise sources and whether mitigation strategies were attempted would be beneficial.

 4. **Lack of In-Depth Electron Transmission Analysis:** The study mentions that electron transmission effects were left for the full-scale setup, yet the border effects and lack of a field shaper could introduce artifacts that impact the interpretation of the GEM extra factor.

 5. **Unexplained Anomalies in Gain Curves:** The non-parallel gain curves suggest complex interactions between the GEM and MM stages, possibly due to backflow effects, but no thorough hypothesis or further investigation is proposed.

 6. **Limited Theoretical Comparison:** While experimental findings are well presented, additional theoretical discussion comparing observed gain trends with expected behavior from simulations or prior literature would strengthen the analysis.

 7. **Scalability Concerns:** The reduction in maximum achievable voltage in the full-scale setup highlights an operational challenge when transitioning from small-scale to large-scale experiments. While this is acknowledged, further discussion on potential solutions or design modifications would be valuable.

 8. **Replication Concerns:** The use of custom-made equipment, electronics, and data acquisition/analysis software (which are not provided or detailed) makes exact replication extremely difficult. Nevertheless, the basic operations, most of the equipment, and parameters are well described, allowing for a similar-but-not-identical replication.

Is the study design appropriate and does the work have academic merit?

Yes

Is the work clearly and accurately presented and does it cite the current literature?

Partly

If applicable, is the statistical analysis and its interpretation appropriate?

I cannot comment. A qualified statistician is required.

Are all the source data underlying the results available to ensure full reproducibility?

Partly

Are the conclusions drawn adequately supported by the results?

Yes

Are sufficient details of methods and analysis provided to allow replication by others?

Partly

Reviewer Expertise:

-MPGD detectors, micromegas, time projection chambers, underground science, low background, dark matter

I confirm that I have read this submission and believe that I have an appropriate level of expertise to confirm that it is of an acceptable scientific standard, however I have significant reservations, as outlined above.

## References

[1] : A Compact Muon Tracker for Dynamic Tomography of Density Based on a Thin Time Projection Chamber with Micromegas Readout. *Particles* .2021;4(3) : 10.3390/particles4030028 333-342 10.3390/particles4030028

[2] : Muon tomography using micromegas detectors: From Archaeology to nuclear safety applications. *Nuclear Instruments and Methods in Physics Research Section A: Accelerators, Spectrometers, Detectors and Associated Equipment* .2019;936: 10.1016/j.nima.2018.10.011 14-17 10.1016/j.nima.2018.10.011

# Response

**Published:** 2025-03-12

**Authors:** Óscar Pérez Lázaro

**Affiliations:** Theoretical Physics, University of Zaragoza Department of Theoretical Physics, Zaragoza, Aragon, Spain

Dear Reviewer, 

 We would like to thank you for your careful reading of our manuscript and for the constructive comments provided. Your suggestions have helped us improve both the clarity and robustness of our presentation. Below, we detail our responses to each of your points: 

 1. **Target Audience and Broader Community**

  We agree with your suggestion regarding broadening the scope of our introduction. In the revised manuscript (Section 1, paragraph 1), we have now added references to recent muography studies and included a statement that highlights the relevance of these detectors not only to the astrophysics community but also to other Micromegas + TPC user communities, such as those in geophysics, civil engineering, and archaeology. This inclusive approach underscores the versatility of Micromegas-based detectors.

 2. **Discussion of Systematic Errors**

  In Section 3.1 (the paragraph beginning “Comparison of the position of the 5.9 keV peak in 55Fe calibration runs…”), we have clarified that the observed ±20% variation in the GEM extra factor arises from slight differences in the highest stable voltages (±5 V in V_mesh and V_GEM), as well as environmental variations (such as temperature and pressure) over several days and across different detectors. We now explicitly state that these variations inherently account for the systematic uncertainties in our measurements.

 3. **Justification for Missing Data and Noise Issues**

  Regarding the absence of the preamplification factor at 1 bar, we have added a comment in the Methods section to explain that the noise issues were traced back to fluctuations in the preamplifier and amplifier modules. We attempted several modifications (such as substituting alternative preamplifier and amplifier modules) but these efforts did not sufficiently reduce the noise. Since the main focus was on the GEM extra factor (which was successfully measured), we proceeded with our analysis while noting that further work on noise reduction is ongoing.

 4. **Electron Transmission Analysis in the Small-Scale Set-up**

  We acknowledge that the small-scale set-up was intended as an intermediate step. In the revised manuscript, we now include a statement clarifying that the small set-up was primarily used to demonstrate the feasibility of preamplification. We have done this by modifying paragraph 4 in Section 3.1: “Here, in all cases, E_drift = 100 V cm⁻¹ bar⁻¹ and E_transfer = 100 V cm⁻¹ bar⁻¹, values which usually lie within the electron transmission plateau of the Micromegas. However, border effects cannot be excluded given the active area dimensions and the absence of a field shaper, so these values might not represent the optimal operating conditions. Nevertheless, given that the purpose of this set-up was just to prove the feasibility and potential of the combined GEM + MM system when installed in real experimental conditions in TREX-DM, detailed field optimizations were not the focus at this stage, and therefore, the in-depth study about the electron transmission (and gain curves) of the GEM + MM was left for the full-scale set-up in Subsection 3.2.” This change highlights that while these values may not be fully optimized, the set-up serves its purpose as a feasibility study.

 5. **Anomalies in Gain Curves and Future Systematic Studies**

  We have noted that the non-parallel gain curves could be related to interactions between the GEM and Micromegas stages, possibly via backflow effects. To address this, we are in the process of constructing a dedicated set-up in Zaragoza (based on a small chamber similar to our current test system) in which we will perform systematic studies using UV laser calibration on a photocathode. Additionally, measurements with the GEM positioned at different distances relative to the Micromegas are planned to further elucidate these interactions. We have added a brief note to this effect in Section 3.2, acknowledging that a more detailed analysis will be the subject of future work.

 6. **Limited Theoretical Comparison**

  We appreciate the suggestion for a more detailed theoretical comparison. Although the current experimental results already provide strong evidence in support of our conclusions, we have now included a comment in Section 3.1 (the paragraph beginning “Comparison of the position…”) about planned theoretical efforts to compare the observed gain trends with expectations from existing models. We believe that a deeper analysis will further enhance the understanding of the underlying processes and is underway. However, even if a complete understanding/modelling of the combined gain curves was not achieved, we still consider the experimental results presented in this article demonstrate the feasibility of preamplification in TREX-DM conditions and constitute an important result on its own.

 7. **Scalability Concerns in the Full-Scale Set-up**

  We recognize the challenge of achieving comparable voltage stability in the full-scale set-up, which is an ongoing area of development. To mitigate these issues, we are exploring the use of resistive Micromegas, which have demonstrated increased stability with respect to spark formation. This approach is expected to yield performance closer to that of the small-scale set-up. We have added a brief comment discussing these efforts in the last paragraph of Section 3.2.

 8. **Replication Concerns and Transparency of Custom-Made Equipment**

  We agree that the use of custom-made equipment and data acquisition software poses challenges for replication, which is sometimes due to certain aspects of our experimental configuration needing very specific requirements (e.g., the number of channels and design constraints). To improve transparency, we have provided more information about the data taking and analysis processes in the small-scale set-up in last paragraph of Section 2.1, and of course we are open to providing more details and helping anyone with an interest in reproducing these results. 

 In summary, we appreciate your detailed comments and believe that the revisions strengthen our manuscript and provide a clearer context for our results. We believe that further improvements and additional studies will only enhance the robustness of our findings. 

 Thank you very much for your valuable input. 

 Sincerely,

 The authors

# Reviewer report: Reviewer response for version 1

**DOI:** 10.21956/openreseurope.20846.r51390

**Published:** 2025-03-04

**Authors:** David Attié

**Affiliations:** 1 Université Paris-Saclay, Gif-Sur-Yvette, France

Dear authors,

 * Summary

 This article deals with a novel approach to enhance energy thresholds in low-background gaseous time projection chambers with high pressure gas mixture (up to 10 bar) using a Micromegas readout plane with an additional GEM preamplification stage.

 The setup aims to improve the maximum effective gain for applications like dark matter searches (i.e. TREX-DM). The results indicate significant extra gain factors in various gas pressures, which help lower detection thresholds. A full-scale TREX-DM setup has been tested to replicate these findings effectively.

 The article demonstrated the feasibility of an amplification scheme using Micromegas+GEM that should allow approaching the single-electron sensitivity in realistic TREX-DM implementations. This amplification scheme (GEM + MM combined) is being installed and commissioned at the TREX-DM experiment.

 The improvement would open a new detection window at lower recoil energies that might lead to substantial improvement to low mass WIMPs.

 * General comments:

 - The article is written in a well-understandable English. 

 - The word Microbulk should be written with the first letter in upper case, same with Mylar, Kapton and X-ray.

 *Minor corrections to be done:

 - §1 Introduction

               > In 3rd paragraph, change "microbulk" to "Microbulk"

               > In 5th paragraph, change "mylar" to "Mylar", add a comma after "At each side," change kapton to Kapton and microbulk to Microbulk. Change "by means of a preamplification" to "by a preamplification" to avoid wordiness.

               > In 6th paragraph, change "microbulk" to "Microbulk" (x3) and "kapton" to "Kapton".

               > In 7th paragraph, change "microbulk" to "Microbulk" (x2). Remove the sentense : "This is also, to our knowledge, the first time that a combined GEM + MM readout is seriously considered for installation in a real experiment.". The hybrid (Microbulk + GEM) detector is used for the first time, but a combined GEM + MM have already been used in a real experiment (see Reference 1 and 2)

               > In 8th paragraph, change "microbulks" to "Microbulks".

 - Caption Figure 2: change "microbulk" to "Microbulk".

 - §2.1

               > In 1st paragraph, change "microbulk" to "Microbulk".

               > In 2nd paragraph, change "microbulk" to "Microbulk", "x-ray" to "X-ray".

               > In 3th paragraph, remove "in order" to avoir wordiness.

 - §2.2

               > In 1st paragraph, change "8 l h-1 is set during 72 h in order to ensure..." to "8 L h-1 is set during 72 h to ensure...". Change "microbulk" to "Microbulk". Change "(256 en each direction)" in "(256 in each direction)". Change "x-ray" to "X-ray".

 - §3.1

               > In 1st paragraph, change "...the GEM with respect to a fixed..." to "...the GEM regarding a fixed...".

               > In 5th paragraph, change "microbulk" to "Microbulk".

 - §3.1

               > In 2nd paragraph, change "...but several hypothesis are..." to "...but several hypotheses are...".

               > In 4th paragraph, change "...such as CH4 or CF4 C4H10 due to their..." to "...such as CH4 or CF4 due to their...".

               > In 6th paragraph, change "...due to some backflow." to "...due to some ion backflow."

 - §5 Conclusions

               > Change "...increase signal to noise ratio..." to "...increase signal-to-noise ratio...".

               > Change "microbulk" to "Microbulk" (2x).

               > Change "In this paper we..." to "In this paper, we...".

               > Change "...potentially down to unexplored region..." to "...potentially down to an unexplored region...".

 - In References

               > 5. Change "Development and performance of microbulk Micromegas detectors" to "Development and performance of Microbulk Micromegas detectors"

 - The Compass experiment has already used an hybrid detector (GEM+Micromegas), but it was with bulk. This paper is the first time using and hybrid with Microbulk.

 A minor revision of this paper has to be done, and comments/corrections have to be considered before indexed.

Is the study design appropriate and does the work have academic merit?

Yes

Is the work clearly and accurately presented and does it cite the current literature?

Partly

If applicable, is the statistical analysis and its interpretation appropriate?

Not applicable

Are all the source data underlying the results available to ensure full reproducibility?

Yes

Are the conclusions drawn adequately supported by the results?

Yes

Are sufficient details of methods and analysis provided to allow replication by others?

Yes

Reviewer Expertise:

Micromegas detector, TPC

I confirm that I have read this submission and believe that I have an appropriate level of expertise to confirm that it is of an acceptable scientific standard, however I have significant reservations, as outlined above.

## References

[1] : Aging effects in the COMPASS hybrid GEM-Micromegas pixelized detectors. *Nuclear Instruments and Methods in Physics Research Section A: Accelerators, Spectrometers, Detectors and Associated Equipment* .2024;1065: 10.1016/j.nima.2024.169511 10.1016/j.nima.2024.169511

[2] : New pixelized Micromegas detector for the COMPASS experiment. *Journal of Instrumentation* .2009;4(12) : 10.1088/1748-0221/4/12/P12004 10.1088/1748-0221/4/12/P12004

# Response

**Published:** 2025-03-12

**Authors:** Óscar Pérez Lázaro

**Affiliations:** Theoretical Physics, University of Zaragoza Department of Theoretical Physics, Zaragoza, Aragon, Spain

Dear Reviewer, Thank you very much for your careful review and constructive comments, they have helped to improve the quality of this article. We have revised the manuscript and incorporated all the suggested corrections. In particular:

- We have updated the terminology through the text (Microbulk, Mylar, Kapton, and X-ray) and we have addressed the typos and recommended stylistic corrections.
- In Section 1 (specifically, the 7th paragraph), we have removed the sentence “This is also, to our knowledge, the first time that a combined GEM + MM readout is seriously considered for installation in a real experiment.” We now clarify that this hybrid configuration “…has been tested in the past [17] and successfully implemented in real experiments such as COMPASS [18, 19] using bulk technology, but this is the first time this is done with a Microbulk Micromegas, at high pressures, and in the context of low-background constraints.” We were not aware that the COMPASS experiment upgrade in 2014-2015 had already used a combined GEM + MM readout (we thought GEMs and MMs had been used separately). We thank you for pointing out this important detail, and we have added the corresponding references.

Thank you again for your input and your time. Sincerely,

 The Authors
